# Supplementary material for: Content-rich biological network constructed by mining PubMed abstracts
Source: BMC Bioinformatics. 2004 Oct 8;5:147. doi: 10.1186/1471-2105-5-147 (PMC528731; doi:10.1186/1471-2105-5-147)
Supplement: Additional File 5 — The original Chilibot query results of the term "long-term potentiation (LTP)" and 22 other terms, limiting the latest references analyzed to the years 1990, 1995, 2000, and 2004. [file 1471-2105-5-147-S5.bz2 › chilibotAdditionalFile5/ltp2000/html/CAMKII.html]

 


**CAMKII** (Input: CAMKII ) 

---


**Google Searches:** Entire Web  | EDU domain only  | PDF files only

.

**External Links:** OMIM |
LocusLink
| Swissprot
| GeneCards

  
**Maps of CAMKII**

Simple
Complete
graph in
radiant
tree
square
layout.

**New Hypothesis !**

**Synonyms** 

- calcium calmodulin dependent protein kinase ii   [PubMed]

- camkii   [PubMed]

**Synopsis**

- Our results suggest that AC activation facilitates LTP induction by suppressing protein phosphatases and enabling a persistent increase in the levels of autophosphorylated **CaMKII**.  J Neurosci, 1999 
  [23]

- In the intact brain, visual experience induces alpha **CaMKII** mRNA polyadenylation and translation, suggesting that this process likely occurs at synapses.  Neuron, 1998 
  [23]

- These results indicate a central role of Ng in the regulation of **CaMKII** activity with decisive influences on synaptic plasticity and spatial learning.  Proc Natl Acad Sci U S A, 2000 
  [23]

- These results show that LTP and **CaMKII** activity drive AMPA Rs to synapses by a mechanism that requires the association between GluR1 and a PDZ domain protein.  Science, 2000 
  [23]

- When the NMDA channels are open, the determinant role in receptor phosphorylation is played by the PKC and **CaMKII**.  Ross Fiziol Zh Im I M Sechenova, 2000 
  [19]

- They suggest a plausible explanation for the very high concentrations of **CaMKII** found in postsynaptic densities of cerebral neurons.  Biophys J, 2000 
  [19]

- Here, we use green fluorescent protein tagged GFP tagged **CaMKII** isoforms and show that CaMKIIbeta is bound to F actin in dendritic spines and cell cortex while CaMKIIalpha is largely a cytosolic enzyme.  Neuron, 1998 
  [16]

- **CaMKII**, and calcineurin, resulting in phosphorylation of AMPA receptors by **CaMKII** and inactivation of NMDA receptors at least in part through calcineurin .  Adv Second Messenger Phosphoprotein Res, 1999 
  [14]

- Here we show that NF L is phosphorylated by **CaMKII** in a subpopulation of apical dendrites during LTP, indicating that Ser 57 is a novel phosphorylation site of NF L in vivo related to the neuronal signal transduction.  J Neurochem, 2000 
  [13]

- Described are methods combining whole cell patch clamp and single cell RT PCR for the detection of a single mRNA species for nitric oxide synthase, or, through a multiplex strategy, for the simultaneous detection of several mRNAs including heme oxygenase 2, protein phosphatase inhibitor 1 protein, and several isoforms of the calcium calmodulin dependent protein kinase II  [**CAMKII**] .  J Chromatogr A, 1998 
  [11]

- In addition to its calcium calmodulin CaM dependent activity, **CaMKII** can undergo autophosphorylation, resulting in CaM independent activity.  Science, 1998 
  [11]

- These results indicate that the cAMP pathway enables TPS induced LTP by inhibiting PP1, thereby enhancing calcium independent **CaMKII** activity.  J Neurosci, 2000 
  [11]

- It is conceivable that a regulatory mechanism such as this may permit **CaMKII** to mediate synaptic frequency encoding and thereby direct an appropriate change in synaptic efficacy.  Neural Comput, 1998 
  [10]

- Internal perfusion of **CaMKII**\*potentiated the maximal effect of kainate.  J Neurophysiol, 1997 
  [10]

- Translocation of **CaMKII** because of interaction with the NMDA receptor calcium channel may potentiate kinase activity and provide exquisite spatial and temporal control of postsynaptic substrate phosphorylation.  J Biol Chem, 1998 
  [10]
